# Assessment of FIV-C infection of cats as a function of treatment with the protease inhibitor, TL-3

**DOI:** 10.1186/1742-4690-1-38

**Published:** 2004-11-19

**Authors:** Sohela de Rozières, Christina H Swan, Dennis A Sheeter, Karen J Clingerman, Ying-Chuan Lin, Salvador Huitron-Resendiz, Steven Henriksen, Bruce E Torbett, John H Elder

**Affiliations:** 1 Department of Molecular Biology, The Scripps Research Institute, La Jolla, USA; 2 Department of Experimental Medicine, The Scripps Research Institute, La Jolla, USA; 3 Department of Animal Resources, The Scripps Research Institute, La Jolla, USA; 4 Department of Neuropharmacology, The Scripps Research Institute, La Jolla, CA, 92037, USA

## Abstract

**Background:**

The protease inhibitor, TL-3, demonstrated broad efficacy *in vitro *against FIV, HIV and SIV (simian immunodeficiency virus), and exhibited very strong protective effects on early neurologic alterations in the CNS of FIV-PPR infected cats. In this study, we analyzed TL-3 efficacy using a highly pathogenic FIV-C isolate, which causes a severe acute phase immunodeficiency syndrome, with high early mortality rates.

**Results:**

Twenty cats were infected with uncloned FIV-C and half were treated with TL-3 while the other half were left untreated. Two uninfected cats were used as controls. The general health and the immunological and virological status of the animals was monitored for eight weeks following infection. All infected animals became viremic independent of TL-3 treatment and seven of 20 FIV-C infected animals developed severe immunodepletive disease in conjunction with significantly (*p *≤ 0.05) higher viral RNA loads as compared to asymptomatic animals. A marked and progressive increase in CD8^+ ^T lymphocytes in animals surviving acute phase infection was noted, which was not evident in symptomatic animals (*p *≤ 0.05). Average viral loads were lower in TL-3 treated animals and of the 6 animals requiring euthanasia, four were from the untreated cohort. At eight weeks post infection, half of the TL-3 treated animals and only one of six untreated animals had viral loads below detection limits. Analysis of protease genes in TL-3 treated animals with higher than average viral loads revealed sequence variations relative to wild type protease. In particular, one mutant, D105G, imparted 5-fold resistance against TL-3 relative to wild type protease.

**Conclusions:**

The findings indicate that the protease inhibitor, TL-3, when administered orally as a monotherapy, did not prevent viremia in cats infected with high dose FIV-C. However, the modest lowering of viral loads with TL-3 treatment, the greater survival rate in symptomatic animals of the treated cohort, and the lower average viral load in TL-3 treated animals at eight weeks post infection is indicative of a therapeutic effect of the compound on virus infection.

## Background

Feline immunodeficiency virus (FIV) is a lentivirus that infects domestic and feral cat populations worldwide. Infected cats exhibit similar disease patterns as human immunodeficiency virus (HIV) infected patients by developing multiple immuno-depletive symptoms collectively referred to as acquired immunodeficiency syndrome (AIDS). As with HIV, differences in virulence among the different FIV subgroups are evident [1-4]. Thus, the cat represents an amenable animal model for testing certain anti-HIV-1 drug modalities *in vivo*.

One of the major breakthroughs in HIV-1 treatment has been the use of specific inhibitors of the viral aspartic protease family as part of a drug cocktail, called highly active anti-retroviral therapy (HAART), with the ultimate goal of suppressing HIV-1 replication in patients to low or undetectable levels [5-8]. Effective HAART therapy continues to be dependent on the development of new drug modalities due to the rapid mutation rate of HIV-1, leading to drug resistance development [9]. Therefore, an effective small animal model for evaluating new drugs and treatments for HIV is of paramount importance. Experimental testing of new protease inhibitors in cats has been of limited success due to the ineffectiveness of HIV-1 specific protease inhibitors against FIV [10,11]. The promising development of the protease inhibitor TL-3, which inhibits FIV, HIV-1 and SIV (simian immunodeficiency virus) infections *in vitro *with similar effectiveness [12] led us to analyze its efficacy in the cat model. Initial *in vivo *studies using the predominantly neurotropic FIV-PPR strain, showed that TL-3 treatment lowered plasma viral loads and resulted in a significant protective effect against neurologic alterations in the CNS in FIV infected cats [13]. In the present study, we employed the highly pathogenic FIV-C isolate (CABCpady00C), which causes a fulminant acute phase disease in the periphery, with high death rates from acute phase immunodeficiency disease [1].

## Results

### In Vivo Infection

Twenty-two female specific pathogen-free (SPF) cats were randomly divided into five groups. Group 0 consisted of two cats, which received TL-3 treatment without viral infection and were considered controls. Group 1 (n = 5) received 0.1 ml (10^5 ^RNA copies/ml) of FIV-C-infected plasma I.V. with TL-3 drug treatment. Group 2 (n = 5) received 0.1 ml FIV-C-infected plasma without TL-3 treatment. Group 3 (n = 5) received 0.5 ml FIV-C-infected plasma with TL-3 and Group 4 (n = 5) received 0.5 ml FIV-C-infected plasma without TL-3 treatment. Blood (1 ml) was drawn from all cats prior to the start of the experiment, at weekly intervals for the first four weeks after infection, and at bi-monthly intervals from week 4 until the end of the study. Complete blood counts were assessed as a function of infection and TL-3 treatment. In addition, quantitative reverse transcription PCR (QRT-PCR) analyses were performed to assess plasma viral load. All animals were continuously observed for any changes in general health. No significant differences were noted between viral load or disease phenotype between the two plasma dosages used in infection and subsequent discussion will not distinguish between these two groups.

By week 6 post infection, seven animals (221, 222, 220, 234, 229, 219, 215) were showing clinical signs of debilitating acute phase disease. Four of the seven affected animals (215, 219, 220, 234) were from the untreated groups, and three animals (222, 221, 229) were from the TL-3 treated groups. Symptoms in all seven symptomatic cats varied from conjunctivitis, anorexia, corneal ulcerations, and gingivitis to increases in temperature, dermatitis and marked lethargy. Despite intensive antibiotic treatment, the general state of health of 6 of the cats did not improve (221, 220, 234, 229, 215, 219) and they received mandated euthanasia between six to seven weeks post-infection. Cat 222 (TL-3 treated) responded to antibiotic therapy and recovered from acute phase symptoms.

**Figure 1:**
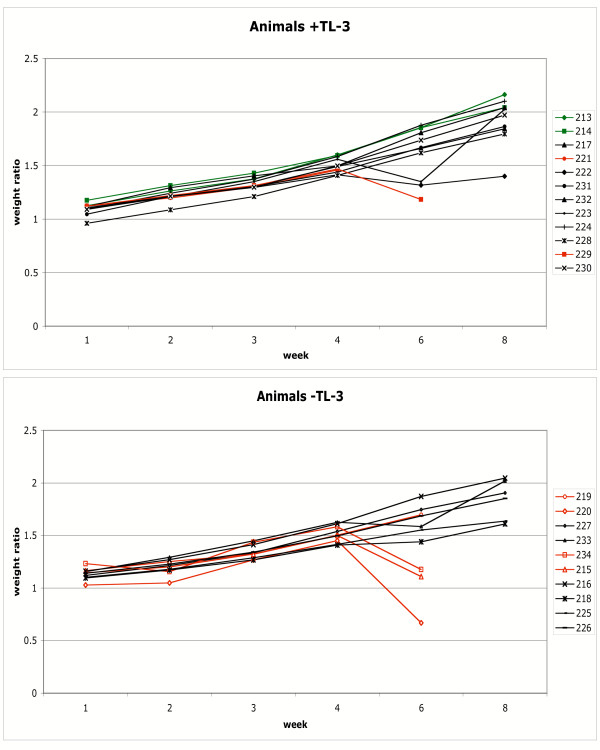
**Cat weight ratios as a function of FIV infection. **Cats infected with FIV-C in the presence and absence of TL-3 treatment were weighed regularly throughout the course of infection. Graphs depict weight of individual cats as a ratio to respective starting weight. Control cats were 213 and 214; green symbols. Weight ratios of cats that required euthanasia as a result of acute phase feline AIDS are shown in red.

Control and infected cats gained weight at approximately the same rate during the first 4 weeks post infection, regardless of drug treatment status (Figure 1, data expressed as a ratio to starting weight for each animal). However, at 6 weeks post infection, three animals in the + TL-3 cohort (Figure 1, upper panel) and three animals in the -TL-3 cohort (lower panel) had lost weight. All three of the animals in the -TL-3 cohort (bottom panel, shown in red; 215, 220, and 234) required mandated euthanasia prior to the next weighing at week 8. Cat 229 in the TL-3 treated cohort also required mandated euthanasia prior to week eight. Cat 221 in the + TL-3 cohort was euthanized on the same day as cat 229, but a final weight was not recorded. Cat 219 of the -TL-3 cohort had a normal weight at week 6, but required euthanasia at week 7, with an approx. 10% weight loss relative to week 6 (data not shown in figure). Thus, weight loss at week 6 occurred with onset of severe acute phase disease. Cat 222 in the TL-3 treated group (upper panel) responded to aggressive rehydration and antibiotic therapy, gained weight, and survived the acute phase. Cat 223 was never noticeably symptomatic and it is unclear why this animal showed a dip in weight at week 6 which it recovered by week 8.

### Brainstem auditory evoked potential changes (BAEPs)

Previous studies using FIV-PPR showed that the isolate induces marked and consistent delays in BAEPs of infected cats and that TL-3 could reverse this effect [13]. We, therefore, analyzed the FIV-C infected animals for similar BAEP delays with or without TL-3 treatment. Animals were analyzed at two-week intervals for the first eight weeks of the experiment. Interestingly, no delays in BAEPs were noted with FIV-C infection (data not shown) in spite of high viral loads in the periphery (see below).

### Viral load quantification

**Figure 2:**
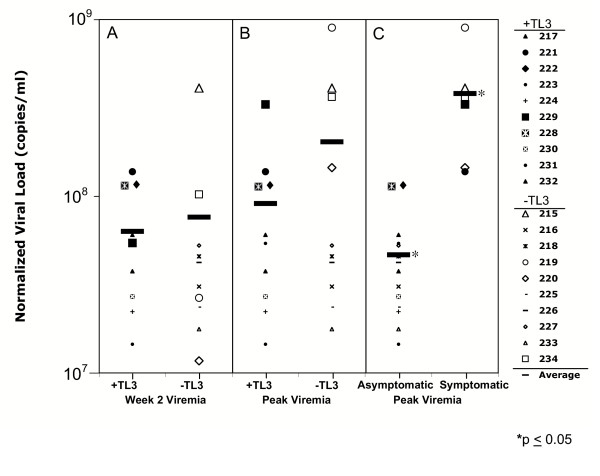
**Plasma viral loads of FIV infected cats as a function of TL-3 treatment and disease. **(A), normalized viral load (copies/ml × 10^6^) at week 2; (B), and at peak viremia between weeks 0 – 6.5 in FIV infected cats treated with TL-3 (+TL-3, solid symbols) or untreated (-TL-3, open symbols). (C), peak viremia between weeks 0 to 6.5 post infection, in healthy (asymptomatic) and symptomatic (euthanized) cats. The average value (largest horizontal bar, –) is plotted for each group. Equal volumes of plasma were normalized using an external RNA spike and analyzed as detailed in Materials and Methods for the presence of FIV RNA by reverse-transcription real-time quantitative PCR. * indicate average values in (C) differ significantly (*p *≤ 0.05).

Plasma viral RNA loads were measured at regular intervals throughout the experimental period to evaluate viral load changes in cats treated with or without TL-3. Viral loads ranged from undetectable to approximately 9 × 10^9 ^RNA copies/ml plasma (Figure 2). Examination of plasma viremia at 2 weeks (i.e. before an active immune response) revealed little differences in viral loads between cats in the TL-3 treated and untreated cohorts (Figure 2A). In contrast, after 2 weeks of infection, the highest viremias were found in cats not receiving TL-3 (compare + TL-3 to -TL-3, Figure 2B). Moreover, of the 6 FIV symptomatic cats that were euthanized due to the severity of clinical disease, 4 of these animals had received no TL-3 and 3 of these cats presented with the highest viral load (compare open symbols to filled symbols, Figure 2C).

**Figure 3:**
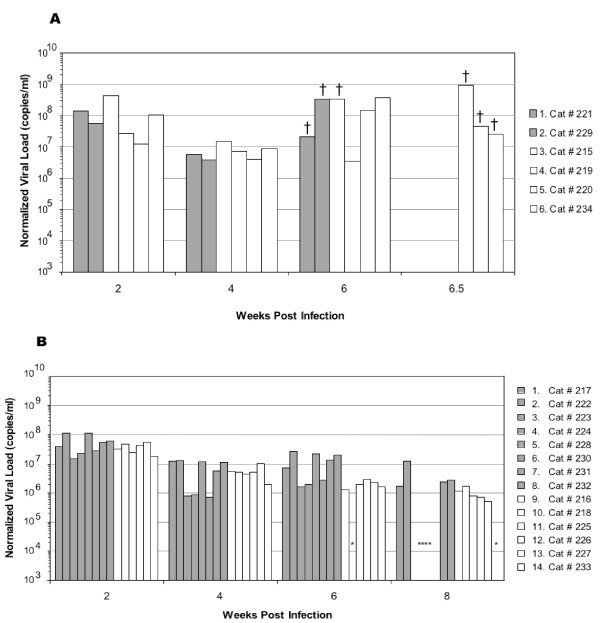
**Individual plasma viral loads as a function of TL-3 treatment over an 8 week period. **Normalized viral loads in (A) symptomatic (euthanized) and (B) asymptomatic cats. Each value corresponds to the volume normalized viral load (copies /ml) between weeks 2–8. Control cats, 213 and 214 (not shown) had viral levels below detection. Cats are grouped for the presence (■ solid bars) or absence (□ open bars) of TL-3 treatment, and then in numerical order from left to right. Specific bars are marked for the approximate week of euthanasia (†) and viral levels below detection are denoted as (*).

Analysis of viremia patterns in individual cats during the initial 8 week evaluation period yielded additional differences in the TL-3 treated vs. untreated groups. Although infected cats showed an initial viral peak in the second week post-infection, by week 4, viral loads decreased for each cat and the average viral load for symptomatic and asymptomatic cats was similar (Figures 3A and 3B). However, by week 6 the symptomatic group had an average viral load of 2 × 10^8 ^copies/ml, 27 times more virus than the average viral load of asymptomatic cats (compare Figures 3A and 3B). Half of the 6 symptomatic cats were euthanized at week 6 due to severe illness. The remaining three symptomatic cats (all from the untreated group) had an average viral load of 3.2 × 10^8 ^copies/ml at 6.5 weeks (Figure 3A) and required euthanasia by week 7. Within the asymptomatic cohort, three (223, 224, 230) out of eight TL-3 treated cats had reduced viral loads by week 4. Their viral loads remained low during week 6 and at week 8, four of 8 surviving TL-3 treated animals had viral load levels below detection limits. Of the six surviving animals that had not received TL-3, only one cat (233) had a viral load below detection limits at week 8 (Figure 3B).

### Leukocyte Changes

**Figure 4:**
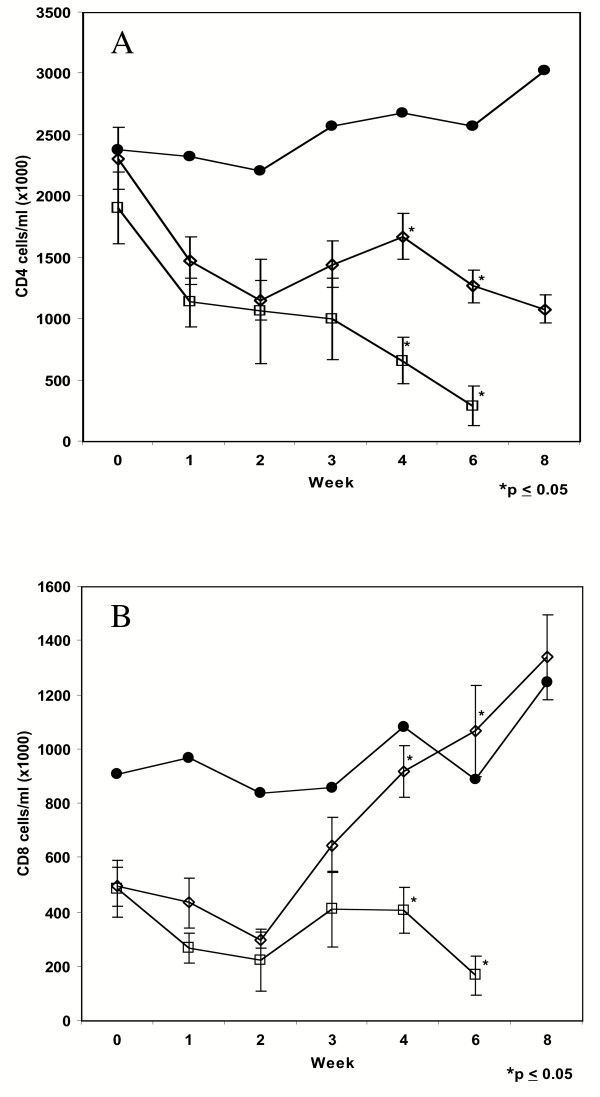
**Peripheral CD4^+ ^and CD8^+ ^lymphocyte levels as a function of clinical outcome over 8 weeks. **A) Total CD4^+ ^cells per ml average and standard error of the mean; B) Total CD8^+ ^cells per ml average and standard error of the mean. Symbols: ●: uninfected cats (213 and 214); □ Symptomatic cats (215, 219–221, 229 and 234); ◇ asymptomatic cats (216–218, 222–228, 230–233). * indicate values between symptomatic and asymptomatic cats differ significantly (*p *≤ 0.05).

Peripheral blood mononuclear cells (PBMC) were isolated from whole EDTA-treated blood from each animal and CD4^+ ^and CD8^+ ^T cell quantitations were performed by flow cytometry and leukocyte counts. Absolute values for CD4^+ ^T cells showed a general decreasing trend in both the TL-3-treated and untreated cohorts when all animals were averaged in each group and CD8^+ ^T cell counts and neutrophils did not show significant variance from control values (data not shown). However, analysis of CD4^+ ^and CD8^+ ^T cells counts of symptomatic cats as compared to treatment-matched asymptomatic animals showed interesting differences (Figure 4A and 4B, resp.). The CD4^+ ^T cell population of the symptomatic cats decreased progressively over the first 6-week period (Figure 4A). CD4^+ ^T cell counts of identically treated cats that showed no signs of disease also decreased, but less precipitously than those of animals eventually requiring euthanasia due to severity of illness (*p *≤ 0.05). Uninfected control animals maintained their T cell values during the same time frame. The CD8^+ ^population of T cells showed an even more drastic decrease in the symptomatic animals compared to the asymptomatic infected animals (Figure 4B). Between weeks two and four, the CD8^+ ^T cell population in the symptomatic animals showed a small rebound from week 2, then declined through week 6 post infection. In contrast, CD8^+ ^T cell counts in treatment-matched asymptomatic animals increased significantly (*p *≤ 0.05) from week 2 onward, consistent with a strong CD8^+ ^T cell response in the protected cats.

Consistent with previous reports [14-16], we also observed changes in the total neutrophil counts in the symptomatic FIV-C infected cats. Within one week post-infection, neutrophil numbers increased markedly in cats 220 (10, 962 cells/μl) and 234 (11, 926 cells/μl) as compared to other cats with identical TL-3 treatment (average = 6880 ± 2847 cells/μl). By week 4, neutrophil values fell drastically in the same two cats (220: 558 cells/μl; 234: 416 cells/μl) as well as in cats 215 (420 cells/μl) and 221 (400 cells/μl). By week 6, four of the symptomatic cats had neutrophil counts near zero. Only cat 220 slightly recovered its neutrophil cell count (3675 cells/μl) prior to mandated euthanasia.

### Protease escape variants

Drug pressure induced viral resistance to protease inhibitors represents one of the major hurdles of HIV HAART treatment regimens [17,18]. HIV and FIV proteases share only 23–28% overall identity at the protein level, yet the enzymatic active site residues are virtually identical [19], allowing a convenient side-by-side comparison. When analyzing the surviving cohort of animals that had received TL-3 treatment, we noted high viral loads in some cats (Figure 3B; 222, 228, 231, 232) that remained elevated (except 228) between weeks 6 and 8, relative to other cats (Figure 3B; 223, 224, 230) that had significantly lowered viral loads. We, therefore, analyzed plasma samples from symptomatic and asymptomatic TL-3 treated animals to look for potential drug-resistance. The protease gene was cloned from week 6 plasma of the two symptomatic TL-3 treated cats (221 and 229) and sequenced. Twenty individual protease clone sequences revealed a wild-type protease gene sequence (data not shown). We then chose cats 231 and 228, two TL-3-treated asymptomatic cats with relatively high viral loads in week 6 (Figure 3B), as well as cat 222, which overcame its disease syndrome with drug treatment. Cloning and sequencing of the protease gene revealed a number of interesting mutations (Table 1). The aspartic acid to glycine point mutation at FIV protease residue 105 (D105G) occurred in cats 231 and 222. The equivalent site in HIV protease is residue 88 (HIV88N), which is associated with development of resistance to some HIV protease inhibitors [20]. Other potentially relevant point mutants cloned from cat 231 were the H72R (HIV63L) and N55D (HIV46M) from week 6 plasma and M107R (HIV90L) from week 8 plasma. The equivalent HIV residue escape mutants exhibit various degrees of resistance against current protease inhibitors [20]. Additional multiple point mutations, lying outside of the FIV protease active site or having no apparent important HIV equivalents were also observed (Table 1). The protease genes were cloned into expression vectors, expressed and purified for enzymatic analysis. The findings revealed that most of the mutants could not be distinguished from wild-type FIV-C protease as to TL-3 susceptibility. However, mutant D105G exhibited a K_i _of 47.3 nM as compared to 9.9 nM for wild-type, consistent with a 5-fold increase in resistance to TL-3.

**Table 1:** FIV Protease Escape Mutants (HIV equivalent residue)

	**PR mutations**	**K_i _vs TL-3 (nM)**
Wildtype PR	----	9.9
Cat 231	D105G (HIV88N)	47.3
	D94G (*)	N.D.
	H72R (HIV63L)	10.9
	N55D (HIV46M)	9.1
	G52R (HIV43K)	N.D.
	^1^M107R (HIV90L)	inactive
	^1^N51Y (HIV42W)	N.D.
Cat 228	C69R (HIV60D)	N.D.
Cat 222	D105G (HIV88N)	47.3

^1 ^isolated from 8^th ^week plasma

* no HIV equivalent site

Bold type, FIV-C point mutant of interest

N.D. not determined

## Discussion

The protease inhibitor, TL-3, demonstrated broad efficacy against FIV, SIV and HIV in tissue culture [12], as well as against drug-resistant HIV isolates [21]. Furthermore, TL-3 treatment had a very strong protective effect on early neurologic alterations in the CNS of FIV-PPR infected cats [13]. However, molecularly cloned FIV-PPR causes little acute phase disease in the periphery. We, therefore, sought to test TL-3 efficacy *in vivo *in the context of the highly pathogenic, uncloned CABCpady00C species (FIV-C) [1], which causes a severe acute phase immunodeficiency syndrome, with high early mortality rates. Although only partial protection was afforded by TL-3 in our studies, the results are promising in that average peak viral loads in some cats were lower in the presence of drug, even in the face of a highly aggressive infection (Figure 2B).

Of 20 cats infected with uncloned FIV-C, seven animals showed signs of immunodepletive disease early on (Figure 4) and developed full-fledged acute phase AIDS symptoms with anorexia, conjunctivitis, corneal ulcerations, gingivitis and marked lethargy by week 6, mandating euthanasia of six animals. The symptomatic cats had viral RNA loads significantly higher (>10^8 ^RNA copies/ml, *p *≤ 0.05) than asymptomatic infected animals independent of drug treatment. This finding suggests that the intense viral infection severely compromised the immune system leading to immunodeficiency and the development of concomitant AIDS, as evidenced by the rapid loss of CD4^+ ^T cells as well as neutrophils in the affected cats during the first few weeks (Figure 4). Cats receiving TL-3 treatment had lower peak viral loads compared to cats not receiving TL-3 at weeks 4 and 8, indicating that the protease inhibitor reduced systemic expansion of viral infection. Previous studies have correlated disease progression with high initial peak viral loads [22]. Of the five out of eight cats treated with TL-3 and having higher viral loads at weeks 4 and 8 compared to non-TL-3-treated cats, three (222, 228, 231) were evaluated for FIV resistance to TL-3. None of the protease genes recovered from cat 228, whose viral levels fell below detection at week 8, showed evidence of TL-3 resistance. However, cats 222 and 231 were found to harbor virus in the plasma that encoded TL-3 resistant protease. In particular, a D105G mutant demonstrated a 5-fold resistance to TL-3 relative to wild type protease, which may indicate the onset of drug resistance development and explain the higher viral levels in some of the TL-3 treated cats. Preparation of isogenic virus containing the D105G point mutation will allow the direct determination of potential drug resistance.

Interestingly, TL-3 treated cats with highest viral loads (221 and 229) developed severe disease syndromes, which suggests that TL-3 efficacy was limited to a specific viral threshold in this study. Once the threshold has been crossed, TL-3 may not be able to overcome the full-blown, acute, viral infection, resulting in rapid onset of immune suppression.

We were unable to show any correlation between humoral antibody responses and clinical outcome (data not shown). However, a consistent observation was a marked and progressive increase of CD8^+ ^T cells in animals surviving the acute phase infection and a lack of such responses in animals that required euthanasia within the first 8 weeks following infection. Although not formally tested, the findings imply a strong cell-mediated response in the surviving animals contributed to controlling the viral infection.

The above analyses underscore the natural variation in the response to FIV challenge in outbred cats, similar to the observed variation in responses to HIV infection in humans. However, more consistent responses, both in peak viral loads (Figure 2) and in lymphocyte counts (Figure 4) were seen when animals were analyzed as a function of disease severity. Thus, the question arises as to whether there is a genotypic link to susceptibility. Upon close scrutiny of the parental heritage pattern, we observed that one male in particular (96AGQ1) had sired eight of the experimental animals with three different females (Table 2). Two of his offspring (213, 214) had been randomly placed into the uninfected control group, while of the remaining six offspring, four (215, 219, 220, 221) succumbed to FIV induced disease. One of the surviving siblings (222) was in the TL-3 treatment group and exhibited conjunctivitis and possible corneal ulceration and was mildly to moderately lethargic. Cat 222 received Baytril treatment and fluid replacement therapy and eventually recovered from her symptoms. The last sibling (216) never showed any detectable signs of disease throughout the experiment. Although complicating the analyses and statistical treatments, the variable responses with FIV infection of cats parallels those observed with HIV infection in humans and thus affords a relevant model for study of infection and treatment modalities. As with humans, cats are outbred, which complicates defining susceptibility markers. However, identifying the genetic basis for susceptibility in the cat may yield important clues to similar phenomena in humans.

**Table 2:** Feline Lineages

**Dam**	**Sire**	**Siblings**			
99AIE3	96AGQ1‡	213*	214*	215†	216◇
00AIB4	96AAK4	217	218		
98AXS5	96AGQ1‡	219†	220†		
00ANU5	96AGQ1‡	221†	222◇		
00AJT2	96ACJ2	224			
01TBK5	99IAS1	225	226	227	
01QAL4	00XAZ4	228			
01QEJ3	98ATY2	229†			
01QBJ3	00IRX4	230			
95PAA3	98IUU1	231			
01IQP4	98IUG3	232	233		
00XBA1	00XAG1	234†			

* control animals

† sacrificed animals

Bold type, offspring from same sire (‡)

◇ survivor offspring of sire

Viral protease inhibitors are of paramount importance for HIV treatment and successful tempering of viral infection. However, drug resistant escape variants are an important consideration in treatment protocols [17,18]. Although we previously failed to isolate TL-3-resistant FIV *in vitro*, the findings here suggest that *in vivo*, drug resistance to the compound may develop. The results were not unexpected in that we had been able to develop TL-3 resistant HIV variants [21] and it seemed unlikely that FIV would prove an exception. The finding of drug resistant mutants, in fact, strongly indicates that the feline/FIV model is valuable in the assessment of the ability of other protease drugs and drug cocktails to suppress virus infection and limit drug resistance development.

## Conclusions

The findings indicate that the protease inhibitor TL-3, when given orally as a monotherapy, did not prevent viremia in cats infected with a high dose challenge with FIV-C and substantial virus loads were evident in circulation throughout the acute phase (between 2–6 weeks post infection) in all infected animals, regardless of drug regimen. Average peak viral loads in the acute phase were lower in TL-3 treated animals, but variability was such that the numbers did not reach statistical significance. However, of six animals that required euthanasia, four were from the untreated cohort and two were from the TL-3 treated group. Additionally, at eight weeks post infection, half the surviving TL-3 treated animals had viral loads below the detection limits, whereas only one of six untreated animals had markedly reduced viral loads. Thus, therapeutic benefit was noted with TL-3 treatment, even in the face of an aggressive FIV infection.

The findings also show clear differences in the lymphocyte responses of animals that succumb to acute phase illness versus those that survive to the asymptomatic phase. The most pronounced difference was in the lack of an increase in CD8^+ ^cell numbers starting around three weeks post infection in animals that eventually required humane euthanasia versus a pronounced and significant increase in CD8^+ ^T cell numbers in animals that survived the acute phase.

Certain animals that received TL-3 had higher than average viral loads after the acute phase. Analyses of the protease genes of FIV quasi-species prevalent in these animals revealed sequence variations relative to protease of wild type FIV-C. One particular protease, cloned and expressed from two TL-3-treated animals, contained the mutation D105G, which imparted 5-fold resistance against TL-3 relative to wild type protease. This may represent the initial stages of drug resistance development and preparation of this mutation in the context of isogenic virus will address this issue. The findings suggest that the cat model will serve as a valuable animal model for study of resistance development against lentivirus infections.

## Materials and methods

### Animals

22 female purpose-bred 8–9 week old kittens purchased from Liberty Laboratories were inspected upon arrival for signs of illness, examined by a veterinarian and weighed. Animals were maintained in a 2-week quarantine and observed for any signs of illness prior to the beginning of the study. IACUC number ARC 61 JAN 3.

### Viral Infection

Plasma samples (10^5 ^RNA copies/ml) from a cat, that had died from an acute infection with CABCpady00C (FIV-C), were kindly provided by Dr. E. Hoover, of Colorado State University. Cats were injected I.V. with either 0.1 ml (10^5 ^RNA copies/ml) or 0.5 ml of plasma.

### Drug Dosing

All procedures for care of cats during dosing as well as dosing procedures were mandated by TSRI's IACUC. Oral TL-3 (L-Iditol,1,2,5,6-tetradeoxy-1,6-diphenyl-2,5-bis [N-[(phenylmethoxy)carbonyl]-L-alanyl-L-valyl]amino]) [12] treatment was initiated in 12 cats, three days prior to infection of ten of the twelve animals with FIV-C. All TL-3 treated animals received 20 mg TL-3 by capsules at eight hour intervals, for approx. the first 7.5 weeks of the experiment. Dosage was then doubled to 40 mg TL-3 per dose at eight hour intervals for an additional week for the two control animals and the eight surviving animals in the TL-3 treated, infected cohort. No adverse effects were noted in the uninfected, TL-3 treated controls.

### Evoked Potentials

Uninfected and FIV-infected animals were intermittently scheduled for analyses of evoked potentials in conjunction with the blood sampling, including testing for both auditory and visual evoked potentials as previously described [23]. Once recordings were complete, a blood sample was collected and animals returned to the vivarium.

### Clinical Evaluation

Animals were examined daily and in case of health concerns further therapy/diagnostics were initiated. Animals with abnormal weight, or on antibiotics were placed on supplemental feeding with moist food and Nutrical. Dehydrated animals received subcutaneous fluid therapy. More affected animals received supportive care and medications, consisting of BID administration of antibiotics (Baytril), BID subcutaneous fluid therapy, BID temperature evaluation, BID application of antibiotic ophthalmic ointment supportive care. Any animals that required extensive supportive care (TID fluid therapy, TID force feeding) were euthanized. Euthanasia of research animals was conducted with strict adherence to NIH Office of Laboratory Animal Welfare protocols.

### Blood Collection and Peripheral Blood Separation

Blood samples (1 ml/animal) were collected weekly during the first month of the study and then every two weeks thereafter, as described [13]. Samples were placed in EDTA blood tubes (1 cc/tube) for further use.

Plasma was separated from blood by centrifugation at 3000 rpm for 5 minutes at room temperature. Blood cells were resuspended in 3 ml PBS and PBMC were separated from buffy coats by density gradient centrifugation using Ficoll-Hypaque Plus (Amersham Biosciences, Sweden). PBMC were washed once in PBS and twice in PBS/2% FBS for flow cytometry analyses.

### Statistical Analysis

Statistical p values for the FIV-C viral load were determined by the Student's two-tailed t-test (paired, two-tailed distribution between the treated and non-treated group and the symptomatic vs asymptomatic group). Statistical p values for the weekly total CD4 and CD8 cell counts were also determined by the Student's two-tailed t-test (paired, two-tailed distribution compared to base line levels at week 0).

### Flow Cytometry Analysis

Two-color flow cytometry analysis was performed on cells stained with mouse α-feline CD4 FITC and mouse α-feline CD8 PE (Southern Biotech, Birmingham, AL). Anti-mouse IgG_1κ _FITC and PE (BD PharMingen, San Diego, CA) were used as isotype controls. Cells were fixed with 2% PFA prior to analysis performed on a FACScan flow cytometer (Beckton Dickenson Immunocytometry Systems) using the Cell Quest Software program.

### RNA Isolation and Reverse Transcription

Plasma for weeks 0, 2, 4, 6 (terminal points for cats 215, 221, and 229), 6.5 (terminal points for cats 219, 220, and 234), and 8, were isolated from whole blood by centrifugation and stored at -20°C. Viral RNA was extracted using the QiaAmp Viral RNA Isolation Kit (Qiagen, Valencia, CA) according to manufacturer's instructions with slight modifications: Plasma samples (280 μl) were lysed in buffer AVL (1,120 μl) (Qiagen) for 10 minutes at room temperature in the presence of carrier RNA (10 μg/ml) and an external Kanamycin (KAN) RNA spike. Equal amounts of the external RNA spike (10^9 ^copies RNA /280 μl plasma), corresponding to the 1.2 kb KAN gene (Promega, Madison, WI), was used to normalize plasma volumes between samples and to correct for sample loss from viral RNA extraction and cDNA synthesis. An on-column DNase/ RNase free (Qiagen) incubation step for 10 minutes at room temperature was added to remove residual cellular DNA. Complimentary DNA (cDNA) was generated in a 20 μl total reaction using 13 μl of sample RNA, 0.5 μl (2 μM stock) each of KAN and FIV specific reverse primers (sequences below) and StrataScript reverse transcriptase, following the manufacturer's protocol (Stratagene, La Jolla, CA). After incubation, each cDNA sample was diluted in water to 30 μl and stored at -80°C for use in real-time PCR.

### Real-Time Quantitative PCR

25 μl real-time PCR reactions were set up containing 2X Platinum Quantitative PCR SuperMix-UDG (12.5 μl) (Invitrogen, Carlsbad, CA), forward, reverse primers and probe mix (7.5 μl), and cDNA target (5 μl). The mixture was incubated at 50°C for 2 minutes, 95°C for 10 minutes, then cycled at 95°C for 15 seconds and 60°C for 60 seconds 55 times, using the ABI Prism 7700 Sequence Detection System (Applied Biosystems, Foster City, CA). Data were analyzed using the ABI 7700 Sequence Detection Software. Forward and reverse primers (10 nM, 100 nM, final concentration respectively) used in real-time PCR were made at IDT, (Coralville, IA), while the fluorescein-dabsyl Amplifluor UniPrimer (100 nM final concentration) was purchased from Serologicals (Norcross, GA). The primer sequences used for real-time PCR are as follows:

FIV reverse-transcriptase forward: 5'-ACTGAACCTGACCGTACAGATAAATTACAGGAA GAACCCCCATA-3'

FIV reverse-transcriptase reverse: 5'-TGTTAATGGATGTAATTCA TAACCCATC-3'

KAN forward: 5'-ACTGAACCTGACCGTACACGCTCAGGCGCAATCAC-3'

KAN reverse: 5'-CCAGCCATTACGCTCGTCAT-3'

### Standard Curves and Background Detection

To determine the relative copy numbers of KAN and FIV from plasma samples, a linear standard curve was generated by plotting 10-fold dilutions (5 × 10^8 ^to 5 × 10^2 ^copies per well) of dsDNA plasmids of known copy number (log scale), against the cycle threshold (Ct) determined for that value. The pET28 vector (Novagen) was used as the target plasmid for the KAN gene, while a plasmid containing the molecular clone of FIV-C was used for the FIV reverse-transcriptase gene. Calculated values for each plasma sample represent *relative *copy numbers for the purposes of evaluation between individual samples.

### Cloning, Purification and Analysis of Protease

Complementary DNA (cDNA) was synthesized from isolated plasma viral RNA of infected cats. The cDNA pool was used as a template for PCR reactions using 5' primer MFIVCPL5' (5'-GATTTATAAATCATATG GCATATAATAAAGTGGGTACCACTACAACATTAG-3'), which adds an NdeI restriction site, methionine, and alkaline to the N-terminal of the protease and 3' primer MFIVCPL33' (5'-CTGAGATCTGAGCAAGCTTTTACATTACTAATCT AATATTAAATTTAACCATG TTATC-3'), which adds a stop codon and a Hind III restriction site to the C-terminus of the protease. The amplified PCR product was gel purified and cloned into pCR-TOPOII vector (Invitrogen, Carlsbad, CA) for sequencing. Selected DNA of mutants, N55D, H72R, D105G, and M107R were digested with Nde I/Hind III and ligated into pET21a expression vector (Novagen). The mutant FIV-C proteases were expressed in Rosetta pLysS cells (Novagen) and purified as previously described [24].

Enzyme kinetics of FIV-C protease were assayed on the flourogenic substrate Arg-Ala-Leu-Thr-Lys(Abz)-Val-Gln/Phe(NO_2_)-Val-Gln-Ser-Lys-Gly-Arg-NH_2_. The concentration was determined by active-site titration with inhibitor TL-3. Inhibitor constant (K_i_) of TL-3 against mutant FIV-C protease was analyzed as described [25].

## Competing Interests

None of the authors have commercial interests or direct association with a company that is marketing TL-3. J.H.E. is a co-author of a patent application regarding use of protease inhibitors like TL-3 reported here, containing small P3 residues, as inhibitors of HIV and FIV.

## Authors' Contributions

S.R. and C.H.S contributed equally to this work and carried out all tissue culture analyses, purification and characterization of cell populations isolated from PBMC, statistical analyses, and preparation of results for publication. Quantitative PCR analyses was carried out by D.A.S. K.J.C. was responsible for all in vivo animal work, including TL-3 administration and oversight of veterinary care for the animals. S.H-R. carried out measurements of brainstem auditory evoked potential changes. S.H., B.E.T, and J.H.E. acted as mentors for the various facets of the project, oversaw the writing of the manuscript, and provided space and funding to carry out the work.
